# An orthogonal system for heterologous expression of actinobacterial lasso peptides in *Streptomyces* hosts

**DOI:** 10.1038/s41598-018-26620-0

**Published:** 2018-05-29

**Authors:** Jimmy Mevaere, Christophe Goulard, Olha Schneider, Olga N. Sekurova, Haiyan Ma, Séverine Zirah, Carlos Afonso, Sylvie Rebuffat, Sergey B. Zotchev, Yanyan Li

**Affiliations:** 1 Laboratory « Molecules of Communication and Adaptation of Microorganisms » (MCAM, UMR 7245 CNRS-MNHN), Sorbonne Universités, Muséum National d’Histoire Naturelle, Centre National de la Recherche Scientifique, CP 54, 57 rue Cuvier, 75005 Paris, France; 2 Department of Biotechnology, Norwegian University of Science and Technology NTNU, N-7491 Trondheim, Norway; 3 Department of Pharmacognosy, University of Vienna, Althanstrasse 14, A-1090 Vienna, Austria; 4 Normandie Université, INSA Rouen, UNIROUEN, CNRS, COBRA, Rouen, France; 5 Present Address: Center for Microalgal Biotechnology and Biofuels, Institute of Hydrobiology, Chinese Academy of Sciences, Wuhan, P. R. China

## Abstract

Lasso peptides are ribosomally synthesized and post-translationally modified peptides produced by bacteria. They are characterized by an unusual lariat-knot structure. Targeted genome scanning revealed a wide diversity of lasso peptides encoded in actinobacterial genomes, but cloning and heterologous expression of these clusters turned out to be problematic. To circumvent this, we developed an orthogonal expression system for heterologous production of actinobacterial lasso peptides in *Streptomyces* hosts based on a newly-identified regulatory circuit from *Actinoalloteichus fjordicus*. Six lasso peptide gene clusters, mainly originating from marine *Actinobacteria*, were chosen for proof-of-concept studies. By varying the *Streptomyces* expression hosts and a small set of culture conditions, three new lasso peptides were successfully produced and characterized by tandem MS. The newly developed expression system thus sets the stage to uncover and bioengineer the chemo-diversity of actinobacterial lasso peptides. Moreover, our data provide some considerations for future bioprospecting efforts for such peptides.

## Introduction

Microbial natural products have been the major source of human medicines and agrochemicals for hundreds of years. Recent advances in DNA sequencing and genomics revealed that microorganisms harbour a wealth of natural product biosynthetic gene clusters, far exceeding the number of known molecules identified via bioactivity-guided screenings. This discrepancy is attributed to the fact that a large number of such gene clusters are not expressed or yield low amounts of products that elude detection under standard laboratory conditions. To enable genomics-based discovery of new natural products, numerous strategies have been developed to activate otherwise cryptic gene clusters, which can be detected and classified using recently developed bioinformatics tools and databases^1–3^. One important approach is to express the pathways in heterologous hosts in combination with cluster engineering or co-expression of a specific regulator^4^.

**Figure 1:**
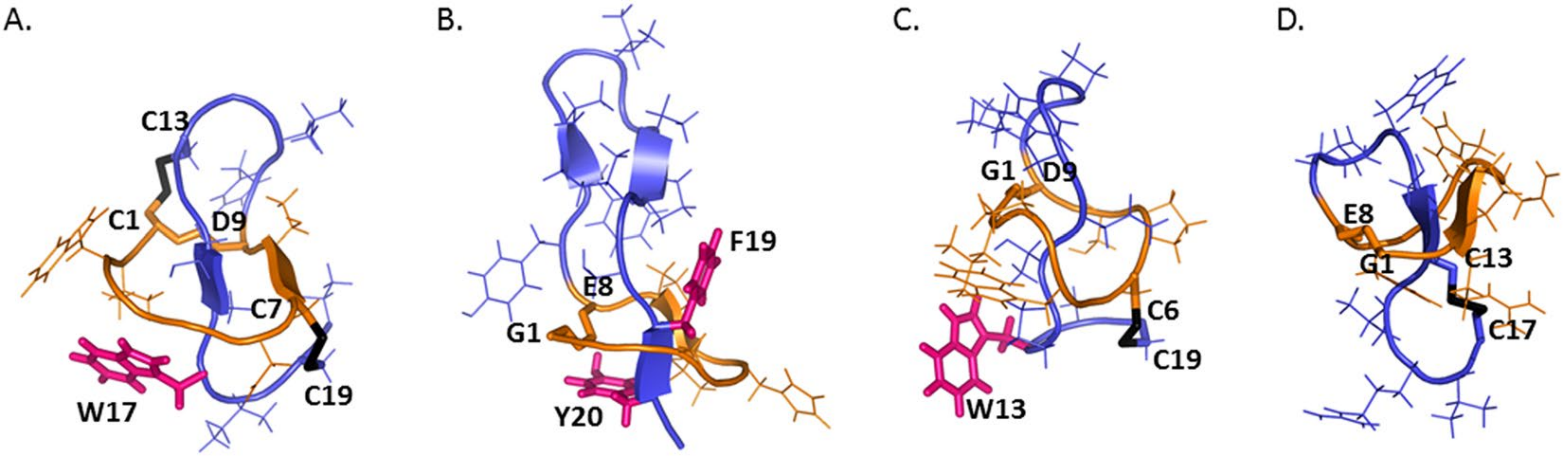
Three-dimensional structures of representative lasso peptides: (**A**) sviceucin (PDB 2LS1, type I). (**B**) microcin J25 (PDB 1S7P, type II). (**C**) BI-32169 (PDB 3NJW, type III). (**D**) LP2006 (PDB 5JPL, type III). Macrolactam ring: yellow; tail: blue, disulphide bridges: black; magenta: bulky residues.

Lasso peptides are an emerging family of ribosomally synthesized and post-translationally modified peptides (RiPPs) produced by bacteria^5^. Composed of 15 to 24 amino acids, they are characterized by an interlocked structure formed by an N-terminal macrolactam ring and a C-terminal tail^6–8^. The 7- to 9-residue macrolactam ring is closed between the amino group of the first residue (Gly, Cys, rarely Ser or Ala) and the carboxyl side chain of a Glu or Asp. The tail threads through the ring; unthreading is prevented by steric hindrance from bulky residues and, in some cases, by additional disulphide linkages (Fig. 1). Lasso peptides are grouped into four classes: those without disulphide bonds form the dominant type II group, whereas the type I and III groups, defined by the presence of two or one disulphide bonds, respectively, comprise very few representatives (two for each type). They display interesting biological activities, such as enzyme inhibition and receptor antagonism, reflecting an advantageous binding of the constrained lasso scaffold to protein targets. Most of the bioactive lasso peptides are isolated from *Actinobacteria*^7,9–16^. Those displaying antibacterial activities are narrow-spectrum and frequently possess novel mode of action, including siamycins that attenuate the expression of virulence factors in *Enterococcus faecalis* by inhibiting a quorum sensing-related histidine kinase^17,18^, lassomycin that displays anti-mycobacterial activity via inhibition of the Clp protease^11^ and streptomonomicin, which potently inhibits the growth of *Bacillus anthracis* by targeting a vital two-component system^12^. These properties thus motivate the search for new lasso peptides from *Actinobacteria*^19^. In this regard, bioprospecting of untapped actinobacterial sources such as those from exotic environments seemed particularly promising in affording bioactive lasso peptides^13,14^.

Genomics-guided approaches greatly accelerated lasso peptides discovery in the recent years. Core lasso peptide biosynthetic genes encode a precursor peptide (A) that encompasses an N-terminal leader and a structural sequence, a cysteine protease (B), a macrolactam synthetase (C) and a RiPP recognition element (E) that binds to the leader sequence^20^. Many clusters alternatively encode fused E-B proteins. Precursor A-^21^ or enzyme B-focused mining^19,22^ of bacterial genomes as well as mass spectrometry-based peptidogenomics^23^ revealed a wide distribution of lasso peptide gene clusters in diverse phyla. Subsequently, a substantial amount of efforts has been made to explore the production potential of *Proteobacteria*, yielding more than 25 new lasso molecules since 2008.^22^ With the exception of microcin J25 (an archetype of lasso peptides produced by *Escherichia coli*), all proteobacterial lasso peptides were produced by expression of the corresponding gene clusters in *E*. *coli*. Heterologous production of lasso peptides is a preferred strategy, owing to the small size of their gene clusters that is amenable to manipulation and that these genes are frequently silent in the native strains under laboratory conditions. Several expression plasmids were developed to allow facile cloning of these clusters, in which transcription of core biosynthetic genes was under the control of an inducible promoter. In combination with genetic engineering and culture optimization, this strategy permitted the production of selected proteobacterial lasso peptides in *E*. *coli* at high yields^24^.

Exploration of *Actinobacteria* for bioactive lasso peptides using genomics-guided approach is emerging, with relatively few examples^14,19,25–27^. Among them, sviceucin is the only case in which high level production was achieved by expressing the cosmid-borne, native cluster in *Streptomyces coelicolor*^26^. Our previous work on other actinobacterial lasso peptides showed that simply transferring the native cluster to *S*. *coelicolor* did not reproducibly lead to peptide production^7^. There is a lack of a general strategy for heterologous expression of actinobacterial lasso peptides, in contrast to those originating from *Proteobacteria*. We report here an effective orthogonal expression system designed to circumvent toxicity problems encountered during cloning of actinobacetrial lasso peptides gene clusters in *E*. *coli*, and thus to produce new molecules in heterologous *Streptomyces* hosts. Our results also provide some guidelines in future bioprospecting efforts in *Actinobacteria* for lasso peptides.

## Results

### Identification of new lasso peptide gene clusters in the genomes of several actinomycetes

**Figure 2:**
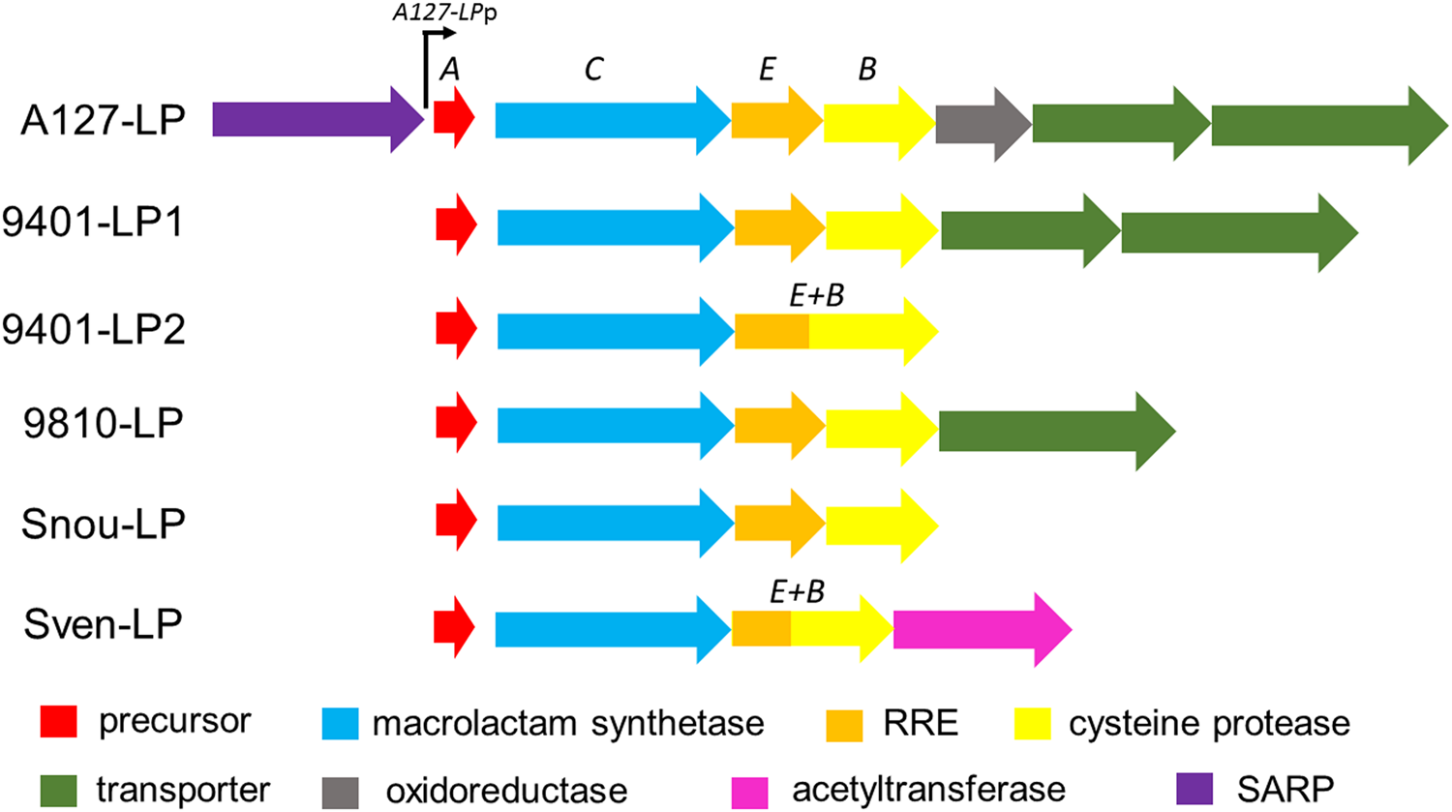
Gene organization of the selected lasso peptide clusters. The core biosynthetic genes (A/C/E/B) are labelled. The SARP and its cognate promoter in the A127-LP cluster used in this study are illustrated. SARP: Streptomyces antibiotic regulatory protein; RRE: RiPP recognition element. A127-LP is from *Actinoalloteichus fjordicus* ADI127-17; 9401-LP1 and 9401-LP2 are from *Streptomyces* sp. ADI94-01; 9810-LP is from *Streptomyces* sp. ADI98-10; Snou-LP is from *Streptomyces noursei* ATCC 11455; Sven-LP is from *Streptomyces venezuelae* ATCC 10712.

Recently-acquired genome sequences from several both known and newly isolated *Actinobacteria* were mined for putative lasso peptide biosynthetic gene clusters. In particular, draft genomes of the following strains were investigated: *Streptomyces* spp. ADI 94-01 and ADI 98-10 (isolated from the marine sponge *Antho dichothoma*, unpublished data), *Actinoalloteichus fjordicus* ADI 127-17 (DSM 46855 ^T^)^28^, *Streptomyces noursei* ATCC 11455, and *Streptomyces venezuelae* ATCC 10712. Using a combination of antiSMASH 3.04^29^ and manual searches, lasso peptides gene clusters were identified in all genomes (ADI 94-01 contained two such clusters). They showed the conserved gene organization following the order *A*-*C*-*E-B* and some contained additional genes involved in the transport, regulation or putative further modifications (Fig. 2). For example, the Sven-LP gene cluster from *S*. *venezuelae* contained a gene encoding an acetyltransferase. The A127-LP gene cluster from *A*. *fjordicus* harboured genes encoding an oxidoreductase as well as a “*Streptomyces* antibiotic regulatory protein” (SARP) (Fig. 2). The primary sequences of the predicted lasso peptides were quite different and diverse, thus representing a considerable chemo-diversity (Table 1). Moreover, they appeared to display new features, including non-canonical residues involved in the macrolactam linkage (the first residue being Ala or Tyr instead of Gly or Cys commonly found in lasso peptides), the presence of one disulphide bond (rare type III lasso peptides) and post-translational modifications decorating the lasso scaffold (Table 1).

**Table 1:** Selected lasso peptide gene clusters.

Name	Precursor peptide sequence	LP size (aa)	LP novel features	Original strain
A127-LP	*MTESLGSYEPPALEEVGIFDEVTL*-**G**RPSWGF**E**ADWSCVMVC	17	type III	*A*. *fjordicus* ADI127-17
9401-LP1	*METNDVYEPPALEEIGGFAELTM*-**A**FGPCVEN**D**WFAGTAWIC	18	type III Ala1	*Streptomyces* sp. ADI94-01
9401-LP2	*MKAGAVPSLEEPAAYEPPMVVDLGHVREVTL*-**G**SSPNGTA**D**ANAQYYY	16	highly hydrophobic	*Streptomyces* sp. ADI94-01
9810-LP	*MKLQKKAYVKPSLFKQGDFSKKTA*-**G**YFVGSYK**E**YWTRRIV	16	Glu9	*Streptomyces* sp. ADI98-10
Snou-LP	*MQGNELQETDKLQTYEAPKMIECGSFQEDTG*-**Y**FGLTGY**E**NLFHFYDKLH	18	Tyr1	*S*. *noursei* ATCC 11455
Sven-LP	*MTDLPRTEEAPAGAEVLDIGDAAELTQ*-**G**QGGGQS**E**DKRRAYNC	16	potential acetylation	*S*. *venezuelae* ATCC 10712

Leader peptides are in italics; residues involved in the macrolactam linkage are in bold; Cys residues involved in the disulphide bond formation are underlined.

### Construction of an orthogonal SARP-based expression system for lasso peptides

In an attempt to generate constructs for heterologous expression, the selected lasso peptide gene clusters were PCR-amplified from the respective genomic DNAs and subjected to Gibson assembly with the pSOK806 vector^30^, placing them under control of a strong constitutive promoter *ermE*p^*^. However, the analysis of recombinant constructs for all the clusters revealed multiple deletions. This suggests a potentially toxic effect of the lasso peptide products arising from gene expression in the cloning host, *E*. *coli*. These cloning attempts were repeated several times, with the same outcome. In order to determine whether the *ermE*p^*^ promoter is active in *E*. *coli*, the plasmid pSOK808^30^ carrying an *ermE*p^*^ promoter::glucuronidase-encoding gene (*gusA*) fusion was introduced into this bacterium, and the GusA activity was measured. A considerable GusA activity could be observed in the crude extracts of *E*. *coli* DH5a carrying pSOK808 (data not shown), confirming that the *ermE*p* promoter is indeed functional in *E*. *coli*.

To prevent expression of the selected lasso peptide gene clusters in *E*. *coli*, an alternative orthogonal two-plasmid system was constructed. We reasoned that promoters from a rare actinobacterium, such as *Actinoalloteichus* sp., will unlikely be recognized by the *E*. *coli* sigma factors. The A127-LP cluster from *A*. *fjordicus* encodes a SARP regulator immediately upstream of the precursor gene A (Fig. 2). It was thus postulated that this SARP controls the expression of A127-LP biosynthetic genes. Several SARPs and their promoters have been characterized in *Streptomyces*^31,32^. Currently it is still impossible to identity the SARP promoter consensus by in silico analysis, especially in an understudied genus such as *Actionalloteichus*. Therefore, the putative promoter region upstream of the precursor gene A was chosen for the experiment. We replaced the *ermE*p^*^ promoter in pSOK808 with the above-mentioned *A127-LP*p promoter and tested the resulting construct, designated pSOK809, in *E*. *coli* for expression of GusA. As expected, almost no GusA activity was detected.

**Figure 3:**
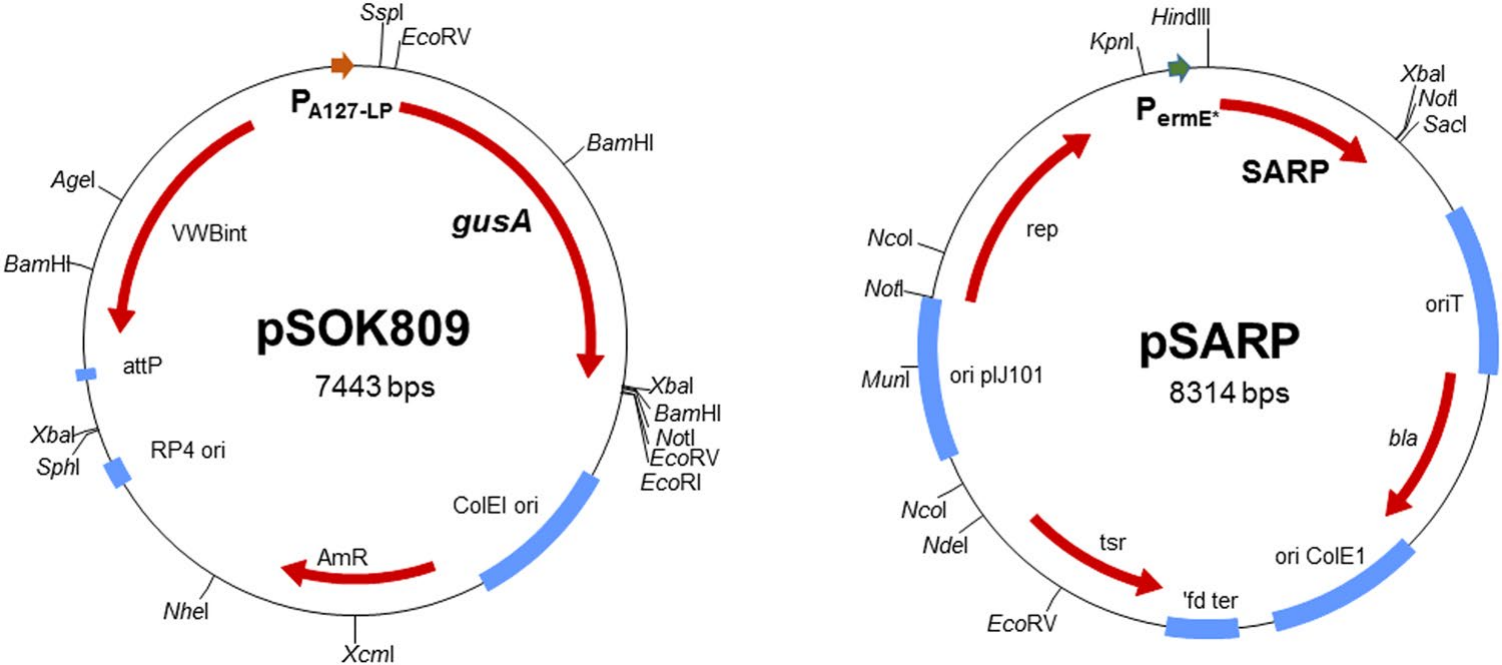
The SARP-based orthogonal two-plasmid expression system.

Next, we introduced the latter plasmid into *S*. *venezuelae*, where rather moderate (*ca*. 15 times lower compared to *ermE*p^*^-based pSOK808) level of GusA activity was observed (data not shown). In order to test our hypothesis about SARP-dependent upregulation of the *A127-LP*p promoter, a multi-copy plasmid pSARP carrying the *A*. *fjordicus* SARP-encoding gene under control of *ermE*p^*^ was constructed and introduced into the *S*. *venezuelae* strain carrying pSOK809. Comparative analysis of the GusA activity in the *S*. *venezuelae* (pSOK809) strains with or without pSARP revealed *ca*. 5 fold higher expression of the *gusA* gene in the latter. Taken together, these results strongly suggested that the developed two-plasmid system (Fig. 3) can be useful for assembly and heterologous expression of lasso peptide biosynthetic gene clusters.

### Heterologous production of lasso peptides in Streptomyces

Indeed, selected lasso peptide gene clusters were successfully assembled in *E*. *coli* under control of the *A127-LP*p promoter using Gibson ligation and pSOK809-based template. The resulting constructs were integrated into the genomes of various heterologous *Streptomyces* hosts via the phage VWB attachment site of pSOK809. Subsequently, the pSARP plasmid was introduced into these recombinant strains. We mainly used *Streptomyces albus* J1074 and *Streptomyces lividans* TK24 as hosts, unless otherwise stated. It was observed that conjugation efficiency for lasso peptide expression plasmids was generally low, implying a potential toxicity problem in *Streptomyces* even at the background expression level without SARP. In particular, no exoconjugants could be obtained for the Sven-LP cluster in both above-mentioned strains, as well as in *S*. *coelicolor* M1146. Conjugation of pA127-LP and pSnou-LP into *S*. *albus* also failed. On the contrary, pA127-LP could be introduced into *S*. *coelicolor*.

For lasso peptide production, recombinant *Streptomyces* strains were grown in four commonly-used rich media and one phosphate-limiting defined medium^33^ in liquid cultures. The latter choice was based on the observation that production of some antimicrobial peptides was triggered by phosphate starvation, e.g. in the case of microcin J25^34^. Liquid chromatography coupled to mass spectrometry (LC-MS) analyses of cell extracts indicated that the best culture condition was MYM or MYM-g medium, under which the expected masses of three peptides (9401-LP1, 9810-LP and Snou-LP) were detected in respective extracts, albeit in trace amount (Table 2).

**Table 2:** Summary of heterologous production of the selected lasso peptides.

LP name	9401-LP1		9810-LP		Snou-LP
Expression strain*	*S*. *albus*	*S*. *lividans*	*S*. *albus*	*S*. *lividans*	*S*. *lividans*
**Liquid medium**					
GYM	ND	ND	ND	ND	ND
MYM	trace	ND	ND	trace	trace
MYM-g	trace	trace	ND	trace	trace
MP5	ND	ND	ND	ND	ND
Pi-limiting defined medium	ND	ND	ND	ND	ND
**Solid medium**					
GYM	ND	trace	trace	ND	ND
SFM	+	trace	**+**	**+**	**++**
ISP2	**+**	trace	trace	ND	trace
ISP4	ND	ND	**+**	ND	trace
Marine Broth	ND	ND	ND	ND	NG
R5	trace	trace	trace	trace	**+**
TSB	ND	ND	ND	ND	ND

^*^Each recombinant strain harboured the corresponding lasso peptide cluster integrated into the chromosome and the replicative pSARP plasmid.

Data not shown in the table: No production was observed for 9401-LP2 (in *S*. *albus* and *S*. *lividans)* and for A127-LP (in *S*. *coelicolor* and *S*. *lividans*).

ND: not detected; NG: no growth; + : promising for scaling-up.

In parallel, seven solid media were tested for lasso peptide production (Table 2). Three out of five expected peptides, 9401-LP1, 9810-LP and Snou-LP, were detected in extracts by LC-MS. They were produced in two or more culture conditions used for the respective strains. The best production medium was specific for each lasso peptide, and media preference did not show a general pattern. No significant differences among expression hosts for either peptide were noticed. It nevertheless appeared, that *S*. *albus* is a slightly better host (Table 2). On the contrary, the peptides A127-LP and 9401-LP2 could not be detected under any tested conditions. The scale-up production and purification was performed for Snou-LP. A yield of 0.8 mg/L solid SFM culture was obtained, a good starting point for further improvement of production.

### Comprehensive characterisation of new lasso peptides by mass spectrometry

**Figure 4:**
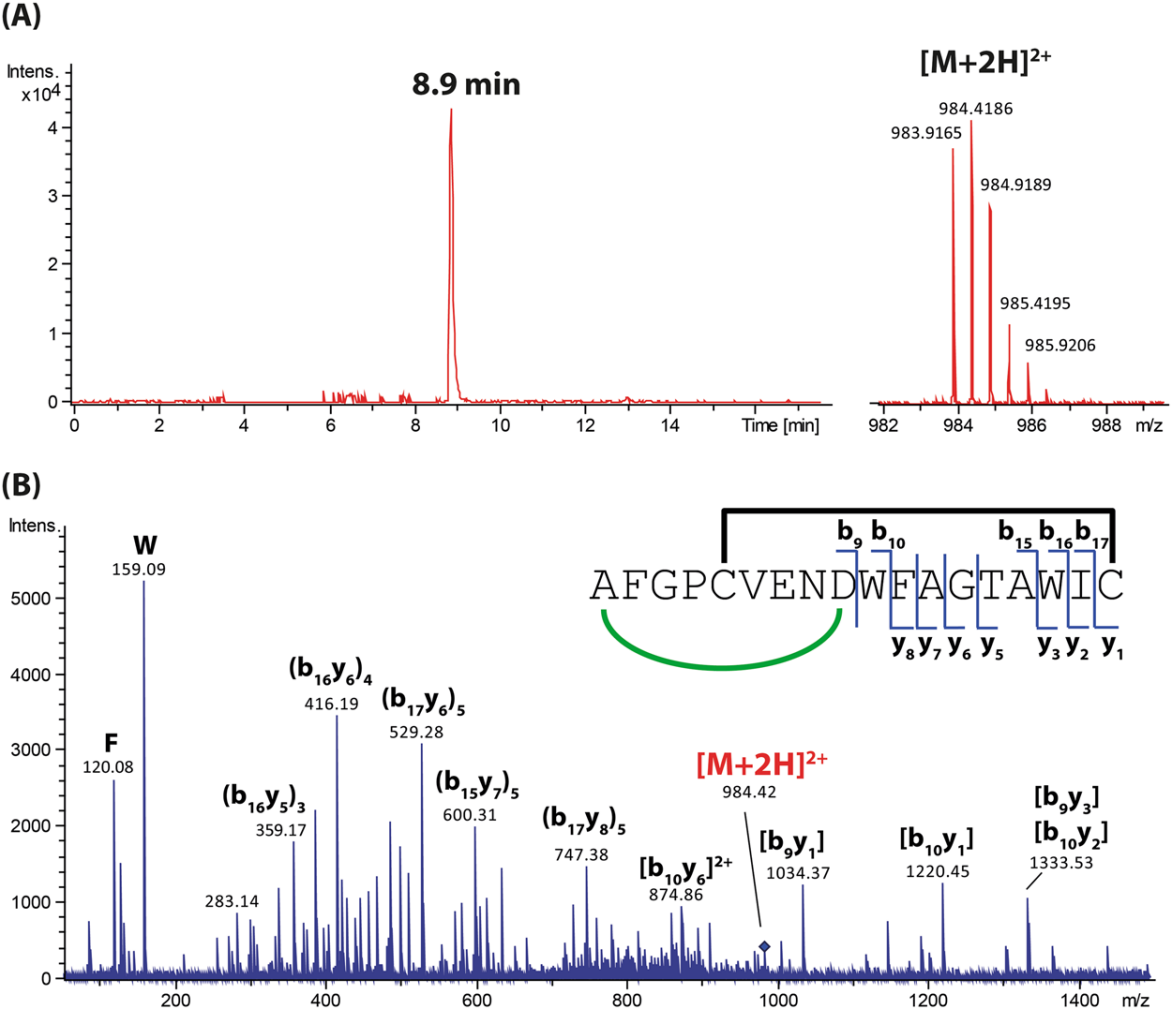
Characterization of the lasso peptide 9401-LP1. (**A**) Extracted ion chromatogram (left) and mass spectrum (right) of the [M + 2 H]^2+^ ion of 9401-LP1 (*m/z* 983.92). (**B**) MS/MS spectrum of the [M + 2 H]^2+^ ion of 9401-LP1 (*m/z* 983.92, collision voltage 40 V).

**Figure 5:**
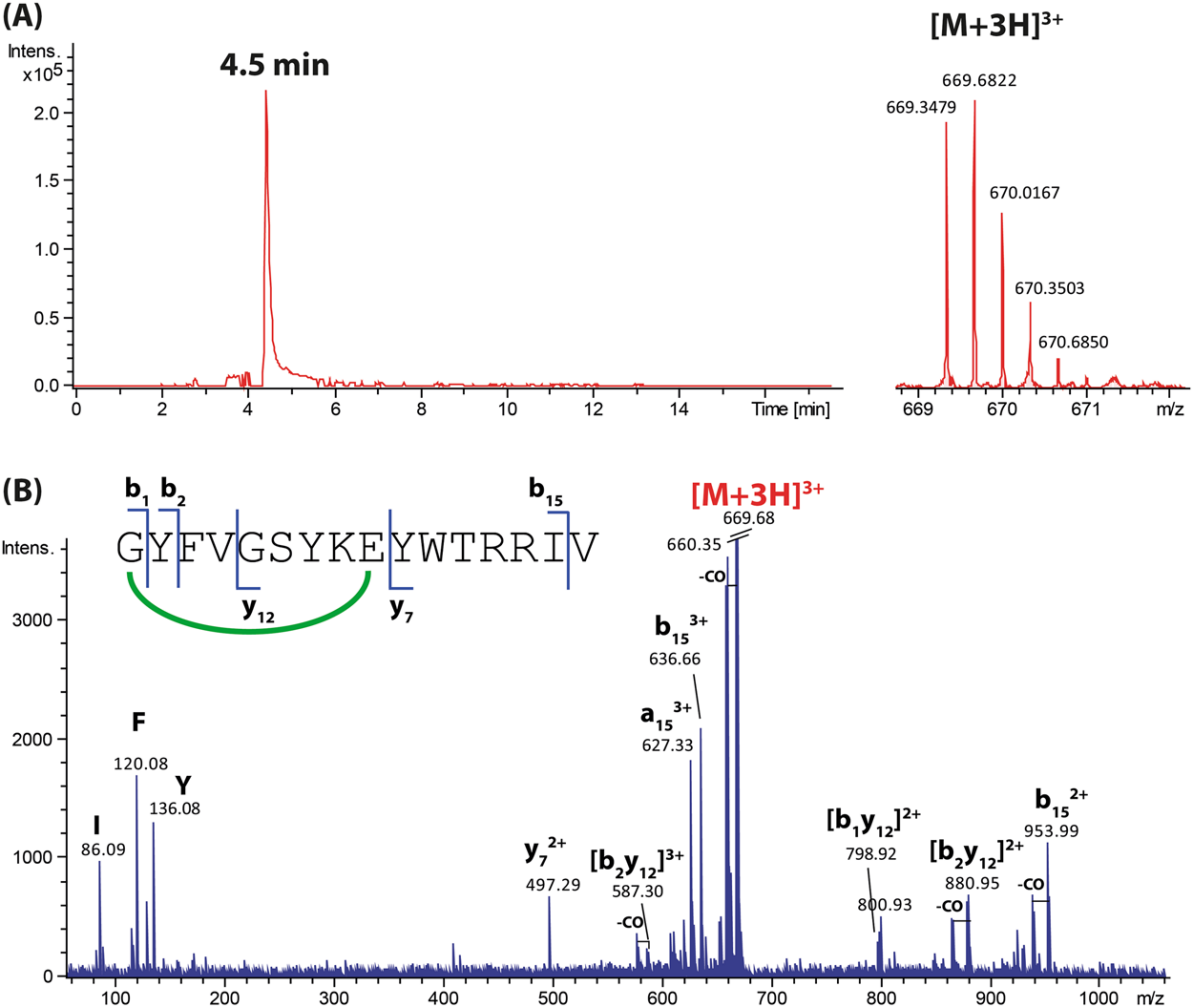
Characterization of the lasso peptide 9810-LP. (**A**) Extracted ion chromatogram (left) and mass spectrum (right) of the [M + 3 H]^3+^ ion of 9810-LP (*m/z* 669.35). (**B**) MS/MS spectrum of the [M + 3 H]^3+^ ion of 9810-LP (*m/z* 669.35, collision voltage 30 V).

**Figure 6:**
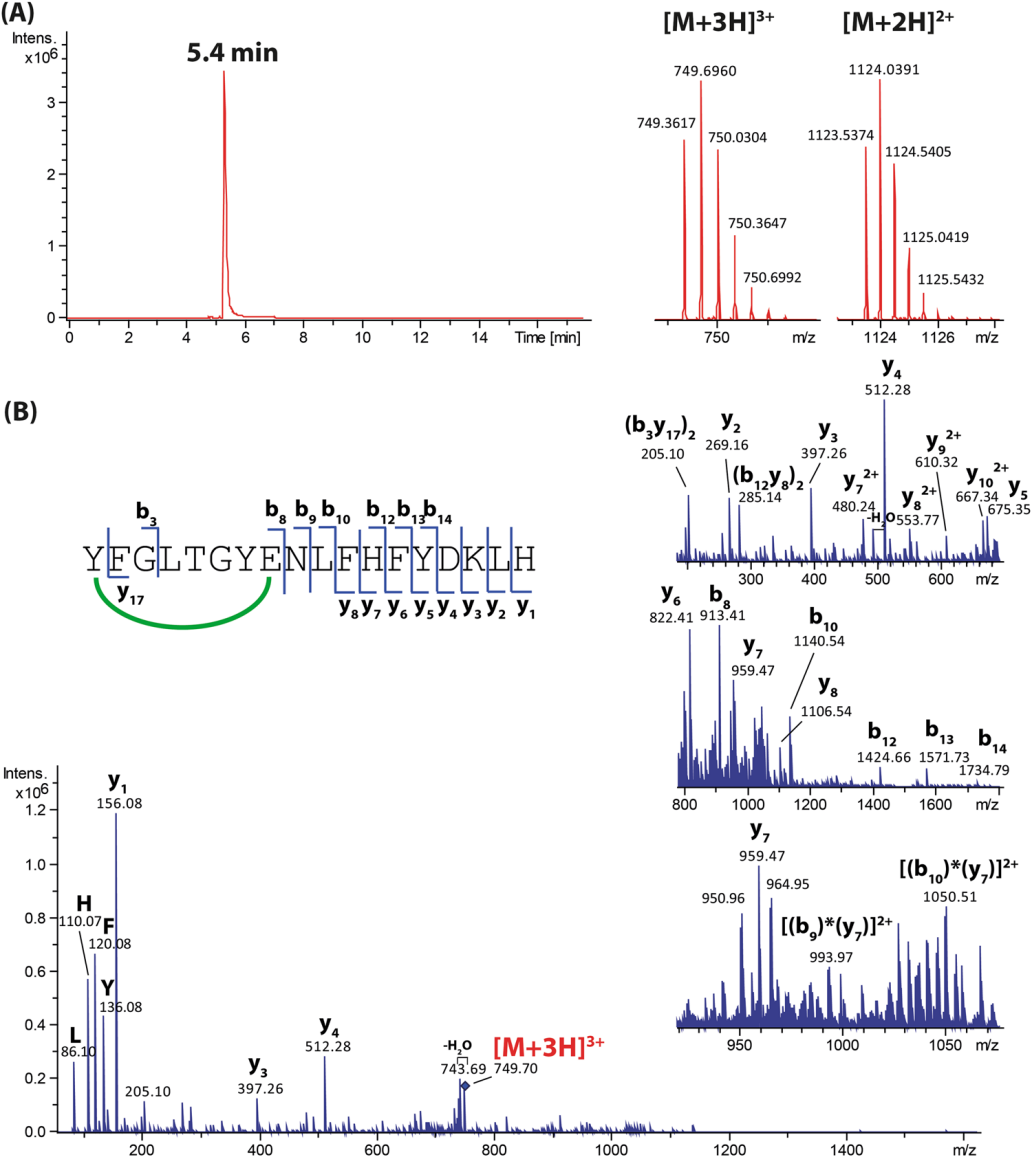
Characterization of the lasso peptide Snou-LP. (**A**) Extracted ion chromatogram (left) and mass spectrum (right) of the [M + 3 H]^3+^ and [M + 2 H]^2+^ ions of Snou-LP (*m/z* 749.36 and 1123.54, respectively). (**B**) MS/MS spectrum of the [M + 3 H]^3+^ ion of Snou-LP (*m/z* 749.36, collision voltage 30 V).

The lasso peptides 9401-LP1, 9810-LP and Snou-LP were eluted at retention times of 8.9, 4.5 and 5.4 min under used LC conditions, respectively (Figs 4–6). The measured *m/z* matched with calculated *m/z* with less than 2 ppm error (Table S4) and the MS/MS spectra matched with the peptide sequences. The MS/MS spectrum of 9401-LP1 revealed mostly fragmentation in the tail regions, yielding b- and y- product ions covalently attached through the disulphide bridge, together with their complementary internal product ions. Moreover, reduction of 9401-LP1 with DTT yielded a mass increase of 2 Da, confirming the presence of one disulphide bond (Figure S1). The peptide 9810-LP appeared more recalcitrant to fragmentation given its high number of basic residues (2 Arg and 1 Lys in the tail and macrolactam ring, respectively). The most abundant fragment ions were b_15_ and y_7_, observed together with product ions resulting from cleavages in the macrolactam ring. Finally, the MS/MS spectrum of Snou-LP appeared more complex, showing many complementary b- and y-type product ions generated by cleavages within the tail region, together with a few [2]rotaxane product ions diagnostic of the lasso structure^35^. The fact that only Snou-LP displayed a MS/MS spectrum characteristic of the lasso topology is not surprising when considering the general tendencies reported on a collection of lasso peptides^36^. For 9401-LP1, the disulphide bridge precludes the formation of the diagnostic [2]rotaxane product ions, while for 9810-LP, these ions may be disfavored due to the short loop region.

The lasso topology was further characterized by carboxypeptidase Y treatment. This exoprotease can cleave branched-cyclic peptides (i.e. non-lasso topoisomers) from the C-terminus up to the amino acid immediately next to the macrolactam ring (see positive control in Figure S2), while lasso peptides are resistant to proteolysis or are hydrolyzed up to the bulky amino acid acting as a plug maintaining the lasso topology^37^. The type III lasso peptide 9401-LP1 was resistant to proteolysis in non-reducing conditions, while it yielded two degradation products upon reduction: [A1-I17] and [A1-W10] (Figure S3). This suggests that in this peptide, the lasso structure is maintained by two factors: the disulphide bridge and a bulky amino acid located at the vicinity of I17 (most probably W16). When the disulphide bridge is reduced, 9401-LP1 appears to become partly unthreaded, thus generating [A1-W10] in the presence of carboxypeptidase Y, while a portion of the peptide still retains the lasso topology, generating [A1-I17]. Peptide 9810-LP yielded several hydrolysis products, namely [G1-I15], [G1-R14] and [G1-Y10] (Figure S4). This suggests that the peptide displays a lasso structure stabilized by amino acids R13-R14 (yielding [G1-I15] and [G1-R14]), but can partially unthread during incubation (yielding [G1-Y10]). Finally, Snou-LP was found to be almost completely resistant to carboxypeptidase Y hydrolysis (Figure S5). Only a minor degradation product [Y1-N9] was observed, suggesting a partial structure unthreading.

The lasso structure of peptides 9401-LP1 and Snou-LP was further confirmed by ion mobility mass spectrometry (IM-MS). This method permits differentiation of lasso peptides from their non-lasso topoisomers based on their difference in collision cross section (CCS, named Ω), especially at high charge states. It has revealed several indicators of the lasso structure, such as the ratio ΔΩ/Ω that measures the relative range of CCS covered by all charge states and the mean charge divided by the molecular mass (named ζ)^38,39^. The IM-MS experiments were carried out in the presence of the sulfolane supercharging agent, in order to favor high charge states. The indicators ΔΩ/Ω and ζ were calculated for 9401-LP1 and Snou-LP (Table S5), and compared with the values obtained for a collection of non-lasso and lasso peptides (including type I, type II and type III representatives). These two indicators were typical of those of lasso peptides for both 9401-LP1 and Snou-LP (Figs S6 and S7).

### Antibacterial activity

None of the new lasso peptides showed activity against tested Gram-negative (*E*. *coli*, *Salmonella enterica*, *Pseudomonas aeruginosa*) and Gram-positive bacteria (*Bacillus megaterium*, *Micrococcus luteus* and *Staphylococcus aureus*). This is consistent with previous reports^11,12,19^, indicating that lasso peptides usually have a very narrow spectrum of activity and hence their target bacteria are difficult to identify.

### Attempts to improve production level by genetic engineering

Although the production of the studied peptides was observed, the yields were unsatisfying. It has been observed that many lasso peptide gene clusters harbour an inverted repeat motif in the intergenic region between the precursor gene A and downstream biosynthetic genes^40^. This terminator-like motif may play a regulatory role in gene transcription. Indeed, modification of this region by an optimized RBS or promoter significantly increased production titers of proteobacterial lasso peptides in *E*. *coli*^24^. Consequently, we employed the same strategy in an attempt to improve the yield of 9401-LP1 as a proof of principle. The 216-bp intergenic region in the 9401-LP1 expression plasmid was replaced by a Kan-*ermE*p^*^ cassette by PCR targeting^41^ so that the transcription of downstream genes was driven by an independent *ermE*p^*^ promoter (Figure S8). This modification led to a 2–4 fold increase of 9401-LP1 production in *S*. *albus*, curiously without the SARP regulator (Figure S9). Results for *S*. *lividans* strains were highly variable and did not give a clear tendency.

## Discussion

Here we report the development of a heterologous expression strategy that allowed the production of three new lasso peptides with a relatively small effort in culture screening. To our knowledge, Snou-LP is the first lasso peptide to have a bulky residue as the first residue (Tyr) and 9401-LP1 represents the third example of the type III group. To date, regulatory mechanisms and factors that trigger lasso peptide biosynthesis in the native producers remain largely unknown. The use of the SARP-responsive promoter *A127-LP*p to drive lasso peptide gene expression in *Streptomyces* bears the advantage of bypassing the native regulatory elements. This strategy is thus applicable to exploit lasso peptide production potential from rare actinobacteria and from metagenomics sources. It also opens a convenient way to generate lasso peptide variants by genetic engineering, facilitating structure-activity relationship studies^37,42^. In addition, our data reveal some aspects that need to be considered in future bioprospecting lasso peptides from *Actinobacteria* by heterologous expression (e.g. toxicity issue, choice of expression hosts etc.).

*S*. *albus* J1074 and *S*. *lividans* TK24 used in this study are well established hosts for heterologous production of secondary metabolites, each being more suitable than the other for certain metabolites^43^. Our data suggest that genotypes of these two hosts do not have a significant influence on lasso peptide production level, at least for the peptides and conditions examined. *S*. *albus* appeared slightly better because lasso peptide production in a wider range of conditions or higher yields were frequently observed for this strain (Table 2). In terms of growth media, solid cultures were clearly found to be superior to the liquid ones for lasso peptide biosynthesis, in consistence with previous studies^23^. However, the most suitable production medium is peptide-dependent and culture optimization of recombinant strains is still necessary. Since *S*. *albus* and *S*. *lividans* are common laboratory strains, the screening process can benefit from the abundant knowledge of their metabolism, and be simplified because of the availability of a range of tested media to choose from.

Modification of the native 9401-LP1 cluster led to a slight improvement of peptide production, showing the necessity of genetic engineering. The knowledge of how the native cluster is regulated will be required for efficient gene cluster refactoring.

Nevertheless, the strategy developed here failed to produce three other lasso peptides (A127-LP, 9401-LP2 and Sven-LP). The gene clusters of A127-LP and 9401-LP2 could be transferred to different *Streptomyces* hosts, and their gene transcription in heterologous hosts was unambiguously confirmed by RT-PCR (Supplementary methods and Figure S10). Therefore, we suspect that the toxicity of the produced peptides to the expression host is a major issue. A precedent example to support this hypothesis is that lariatin, a lasso peptide produced by *Rhodococcus jostii*, could not be produced by expressing the corresponding gene cluster in *Rhodococcus* strains that are closely related to the producer^42^. Several cellular targets of antibacterial lasso peptides have been identified^11,18,44^. In particular, the RNA polymerase has been shown to be a common target of several lasso peptides including microcin J25,^44^ siamycins and capistruin^45^, despite their different antibacterial spectra. The immunity mechanism of the native producer to its toxic lasso peptide is conferred by a dedicated ABC transporter, often encoded in the gene cluster. The striking difference in production between 9401-LP1 and 9401-LP2 clearly indicated that the lack of a resistance mechanism in the heterologous host could account for the failure of 9401-LP2 synthesis, as its gene cluster does not encode a pathway-specific transporter in contrast to that of 9401-LP1. For A127-LP, although transporter genes are present, it is likely that *Streptomyces* strains cannot install effectively the immunity mechanism originating from a phylogenetically distant actinobacterium. Similar phenomenon was observed when expressing the gene cluster of microbisporicin originating from *Microbispora coralline* in *Streptomyces*^46^. Worth of note, Snou-LP could be produced although no transporter genes were found in the cluster, suggesting that its cellular target might be different to those for 9401-LP2 and A127-LP. It is remarkable that the Sven-LP biosynthetic genes could not be conjugated into *Streptomyces*. One peculiarity of this cluster, in addition to the lack of transporter genes, is the presence of a gene whose product shows similarity to ribosomal protein serine acetyltansferases. A precedent example showed that genes encoding ribosomal proteins prevented the conjugation of the gene cluster of GE2270, a thiopeptide identified in *Planobispora rosea*, in *Streptomyces*^47^. It is therefore likely that this gene involved in protein acetylation plays a role in the conjugation process, or its expression is toxic to the host. An alternative explanation for failure to produce lasso peptides heterologously could be the absence in the new host of an unknown factor critical for maturation of the peptide. Overall, our data indicate that the success of heterologous expression is mainly determined by the intrinsic properties of the lasso peptide cluster of interest.

In conclusion, this study developed a SARP-controlled orthogonal expression system for the production of actinobacterial lasso peptides in *Streptomyces*. It is suitable for lasso peptide bioprospecting from yet untapped actinobacteria, and opens a convenient way to produce and engineer lasso peptides with new features that could hold promise for peptide-based drug discovery.

## Methods

### Bacterial strains, plasmids and growth conditions

Bacterial strains and plasmids are listed in Table S1. *Streptomyces* strains are routinely maintained on Soya-Flour-Mannitol (SFM) agar plates. For lasso peptide production, recombinant *Streptomyces* strains were screened in liquid media including GYM (4 g/L glucose, 4 g/L yeast extract and 10 g/L malt extract), MYM (4 g/L maltose, 4 g/L yeast extract, 10 g/L malt extract, 1.9 g/L MOPS), MYM-g in which maltose was replaced by 24 mL/L glycerol, MP5 (7 g/L yeast extract, 5 g/L NaCl, 1 g/L NaNO_3_, 36 ml/L glycerol, 20.9 g/L MOPS, pH 7.5) and phosphate-limited defined medium in which glucose was replaced with 24 mL/L glycerol^33^. Solid media used for lasso peptide production were GYM, SFM, ISP2 (BD Difco™), ISP4 (BD Difco™), marine broth (Pronadisa), R5^48^ and Tryptic Soy Broth (TSB, BD Bacto™) agar. Conjugation of recombinant plasmids into *Streptomyces* was performed as previously described by Flett *et al*.^49^. Antibiotics used were apramycin (50 µg/mL) and thiostrepton (17 µg/mL) for selection of recombinant *Streptomyces* strains.

### Construction and testing of SARP-based expression system

The pSOK809 reporter plasmid was constructed from PCR-amplified fragments using Gibson assembly^50^. DNA fragment encompassing the vector part with *gusA* gene was amplified from the pSOK808 template^19^ using primers 808GUS-F and 808GUS-R (Table S2). Putative SARP-dependent promoter region from the *Actinoalloteichus* sp. ADI127-17 lasso peptide gene cluster upstream of the A gene was amplified from the genomic DNA of the organism using primers SARp-F and SARp-R (Table S2). The pSARP plasmid for expression of the SARP regulator was constructed by a similar procedure. The vector part was amplified from the pUWLoriT template^51^ using primers UWLS-F and UWLS-R (Table S2). The SARP-encoding gene was amplified from the genomic DNA of the *Actinoalloteichus* sp. ADI127-17 using primers SARPg-F and SARPg-R (Table S2). Both plasmids were introduced in *S*. *venezuelae* ATCC 10712 via conjugation from *E*. *coli* ET12567/pUZ8002 either alone or in combination. The resulting strains were tested for GusA activity after 48 h of fermentation in the MYM liquid medium as described in Sekurova *et al*.^30^.

Gibson assembly was also used to construct all the plasmids for lasso peptide expression using the pSOK809 part without the *gusA* gene as a backbone and PCR-amplified genomic fragments encompassing respective gene clusters. Oligonucleotide primers used in construction of recombinant plasmids are listed in Table S2. Sequences of the lasso peptide gene clusters in this study were deposited in the GenBank. Accession numbers are KX379996 for 9401-LP1, KX379997 for 9401-LP2, KX379998 for 9810-LP, KX379999 for A127-LP, KX380000 for Snou-LP and KX380001 for Sven-LP (Table S3).

Oligonucleotide primers were designed using software j5^52^ and Clone Manager 9 (Sci-Ed Software, USA). All constructs were sequenced to verify that no mutations were introduced during PCR amplification.

### Production and extraction of lasso peptides

The SARP-based lasso peptide expression plasmids were introduced into *Streptomyces* strains by conjugation with *E*. *coli* ET12567/pUZ8002 as donor strain. Exconjugants were selected on SFM agar supplemented with 10 mM MgCl_2_ and apramycin. Integration of the gene cluster into the chromosome was confirmed by PCR. The pSARP plasmid was subsequently transferred to the resulting recombinant strain in a similar manner by conjugation. Exconjugants were selected with thiostrepton supplemented in the medium.

Growth on liquid medium for production: A seed culture of each recombinant *Streptomyces* strains harbouring the lasso peptide cluster was grown in the liquid medium of choice supplemented with apramycin and thiostrepton at 30 °C for 2 days. This was used to inoculate 50 mL of the same medium and the culture was grown in the presence of 5% XAD-16 resin at 30 °C for 7 days. The mycelia and the resin were extracted with MeOH at room temperature for 3 hours. The extracts were dried in a rotary evaporator and re-suspended in 60% acetonitrile before LC-MS analysis.

Growth on solid medium for production: A seed culture of each recombinant *Streptomyces* strains harbouring the lasso peptide cluster was grown in liquid GYM medium supplemented with apramycin and thiostrepton at 30 °C for 2 days. This seed culture was spread on the solid medium of choice. The plates were sealed and incubated at 30 °C for 7 days. Next, the agar with mycelia were cut into pieces and extracted in 100% MeOH at room temperature for 3 hours. The debris were removed by filtration; the resulting extracts were dried and resuspended in 60% acetonitrile. To scale up, selected strains were grown in square Petri dishes (10 cm × 10 cm) and extraction was performed as described above. Peptides were enriched from the crude extracts by solid phase extraction (SPE) using a Waters C18 SPE cartridge. 9401-LP1 and 9810-LP were produced in *S*. *albus* on ISP2 and ISP4 plates, respectively, and Snou-LP was produced in *S*. *lividans* on SFM agar.

### LC-MS and MS/MS analysis

SPE fractions were analyzed by LC-MS and LC-MS/MS on an ultra-high performance LC system (Ultimate 3000 RSLC, Thermo Scientific) connected to a high-resolution electrospray ionization – quadrupole – time of flight (ESI-Q-TOF) mass spectrometer (Maxis II ETD, Bruker Daltonics). Separation was achieved on an Acclaim RSLC Polar Advantage II column (2.2 μm, 2.1 × 100 mm, Thermo Scientific) at a flow rate of 300 μL/min, using the following gradient of solvent A (ultra-pure water/0.1% formic acid) and solvent B (HPLC-MS grade acetonitrile/0.08% formic acid) over a total run time of 17.5 min: linear increase from 10% B to 60% B for 12 min, linear increase to 100% B for 0.2 min, decrease to 10% B for 0.5 min. The ESI-Q-TOF instrument was externally calibrated before each run using a sodium formate solution consisting of 10 mM sodium hydroxide in isopropanol/0.2% formic acid (1:1, v/v). The MS data were acquired in positive ion mode in the mass range *m/z* 60–2000. The source parameters were as follows: nebulizer gas 35 psi, dry gas 8 L/min, capillary voltage 3500 V, end plate offset 500 V, temperature 200 °C. LC-MS/MS data were acquired in the multiple reaction monitoring (MRM) mode with selection of the [M + 2 H]^2+^ or [M + 3 H]^3+^ ions of the lasso peptides. The data were treated with Data Analysis 4.3 (Bruker Daltonics). Relative peptide production level was measured by integrating mass peaks corresponding to all charge states.

The nomenclature introduced by Roepstorff and Fohlman^53^, further modified by Biemann^54^, was adopted to describe the product ions generated by MS/MS. The internal fragment ions were thus noted (b_r_y_s_)_(r+s−t)_, where r, s, and t indicate the number of amino acids cleaved counting from the N-terminus, the number of amino acids cleaved from the C-terminus and the total number of amino acid residues in the peptide, respectively. The subscript (r + s − t) accounts for the number of residues in the internal fragment. When the b- and y-type product ions remained associated due to a covalent link (either the isopeptidic bond forming the macrolactam ring or a disulphide bridge), we used the nomenclature [b_r_y_s_]. As an example, [9401-LP1 - {FAGTAWI}] was denoted [b_10_y_1_] (Fig. 4). Finally, the b- and y- type ions associated non-covalently due to the steric entrapping of the tail within the ring were named using the nomenclature [(b_r_)*(y_s_)] introduced previously by our group^35^. These [2]rotaxane product ions detected for certain lasso peptides constitute a signature of the lasso topology.

### Carboxypeptidase Y treatment

The pre-purified peptides 9401-LP1, 9810-LP and Snou-LP were submitted to carboxypeptidase Y hydrolysis, as previously described^22^. Briefly, carboxypeptidase Y from *Saccharomyces cerevisiae* (Sigma) was added at 0.1 U/mL to the peptide solution in MES buffer (50 mM MES, 1 mM CaCl_2_, pH 6.7) and the solution was incubated for 3 h at 37 °C. Negative control consisted of the peptides with no enzyme. Positive control consisted of the non-lasso topoisomer of microcin J25. In case of reduction of 9401-LP1, 50 mM DTT was added before carboxypeptidase Y treatment.

### Ion mobility mass spectrometry

IM-MS experiments were carried out on a hybrid quadrupole ESI-Q-TOF IM-MS instrument (Synapt G2, Waters). The pre-purified peptides 9401-LP1 and Snou-LP, as well as the lasso and non-lasso peptide controls, MccJ25 and MccJ25-lcm, were solubilized in H_2_O/CH_3_CN 1:1 + 0.1% formic acid +0.5% sulfolane. The solutions were introduced in the source by direct infusion using a syringe pump with a flow rate of 300 μL/h. Ionization was operated in positive ion mode under the experimental set-up as described^39^. The CCS calibration was carried out from the CCS values of doubly-protonated polyalanine ions determined with uniform field drift tube ion mobility with helium as drift gas^55^. The 9810-LP was in too low quantity/purity to provide information on the peptide topology by IM-MS.

### Antibacterial activity assays

The antibacterial activity of the new lasso peptides were tested with pre-purified peptides using the soft-agar overlay technique.

### Data Availability

The datasets generated during and/or analysed during the current study are available from the corresponding author on reasonable request.

## Electronic supplementary material

Supplementary Information
