# Supplementary material for: An orthogonal system for heterologous expression of actinobacterial lasso peptides in Streptomyces hosts
Source: Sci Rep. 2018 May 29;8:8232. doi: 10.1038/s41598-018-26620-0 (PMC5974421; doi:10.1038/s41598-018-26620-0)
Supplement: Supplementary file 1 — Supplementary Information [file 41598_2018_26620_MOESM1_ESM.pdf]

Supplementary Information

An orthogonal system for heterologous expression of actinobacterial lasso peptides in *Streptomyces* hosts

Jimmy Mevaere,[a,ǁ] Christophe Goulard,[a,ǁ] Olha Schneider,[b] Olga N. Sekurova,[c] Haiyan Ma,[a,d] Séverine Zirah,[a] Carlos Afonso,[e] Sylvie Rebuffat,[a] Sergey B. Zotchev [c,*] Yanyan Li [a,*]

[a]Laboratory « Molecules of Communication and Adaptation of Microorganisms » (MCAM, UMR 7245 CNRS-MNHN), Sorbonne Universités, Muséum National d'Histoire Naturelle, Centre National de la Recherche Scientifique, CP 54, 57 rue Cuvier 75005, Paris, France.

[b]Department of Biotechnology, Norwegian University of Science and Technology NTNU, N-7491 Trondheim, Norway

[c]Department of Pharmacognosy, University of Vienna, Althanstrasse 14, A-1090 Vienna, Austria

[d]Current address: Center for Microalgal Biotechnology and Biofuels, Institute of Hydrobiology, Chinese Academy of Sciences, Wuhan, P. R. China

[e] Normandie Université, INSA Rouen, UNIROUEN, CNRS, COBRA, Rouen, France

Supplementary Methods:

Gene expression study by RT-PCR: *Streptomyces lividans* strains harboring the gene cluster A127-LP and p9401-LP2 were grown in GYM medium at 30 °C for 4 days. Mycelia from 1 mL culture were recovered and re-suspended in 1 mL Trizol reagent (Invitrogen). Cells were then lysed on a FastPrep-24™ 5G homogenizer (MP Biomedicals) using the lysing matrix B. The total RNAs were subsequently precipitated following the Trizol manufacturer's protocol and further purified using the RNeasy plus mini kit (Qiagen). The eluted RNA was treated with DNase I (Ambion). Synthesis of cDNA was performed with 1 µg RNA, random hexamer and SuperScript III reverse transcriptase (Invitrogen) according to the manufacturer's protocol. Expression of the A and C genes with the house-keeping gene *hrdB* encoding the RNA polymerase sigma factor was subsequently analyzed by PCR using *Taq* DNA polymerase (VWR).

Table S1. Plasmids and strains used in this study.

**Name**	**Description**	**Origin**
**Strains**		
E. coli 12567/pUZ8002	*dam dcm hsdM hsdS hsdR cat tet;* with plasmid pUZ8002; for conjugation	Gust et al. 2003
E. coli DH10b	For plasmid propagation	Invitrogen
S. coelicolor M1146	Δ*act*, Δ*red*, Δ*cpk*, Δ*cda*	Gomez-Escribano and Bibb, 2011

S. lividans TK24	Heterologous expression host	Rückert C et al. 2015
S. albus J1074	Heterologous expression host	Chater and Wilde, 1976
**Plasmids**		
pSOK809	Reporter plasmid to probe the activity *A127-LP*p	This study
pSARP	Expression plasmid for SARP regulator under the control of P_{ermE*}	This study
p9401-LP1	Expression plasmid of 9401-LP1 cluster	This study
p9401-LP2	Expression plasmid of 9401-LP2 cluster	This study
pA127-LP	Expression plasmid of A127-LP cluster with SARP encoded	This study
p9810-LP	Expression plasmid of 9810-LP cluster	This study
pSnou-LP	Expression plasmid of Snou-LP cluster	This study
pSven-LP	Expression plasmid of Sven-LP cluster	This study

Table S2. Primers used in this study.

Name	Nucleotide sequence	DNA template	Final construct
808GUS-F	GGTGTCTGCTGTCCATCGATCGAAGGAGAGTTCACCATGCTGA	pSOK808	pSOK809
808GUS-R	CGAAGCGCCGAACCACGAAGTGCGAAGTTCACCGAAGAGCG	pSOK808	pSOK809
SARp-F	TTCGCACTTCGTGGTTCGGCGCTTCGGTGAACTCCG	*A.fjordicus* ADI127-17 gDNA	pSOK809
SARp-R	ACTCTCCTTCGATCGATGGACAGCAGACACCAGGTCGCCGC	*A. fjordicus* ADI127-17 gDNA	pSOK809
UWLS-F	GGTGCAGCCGAATGCGTATGGTGCACTCTCAGTACAATCTGCTCT	pUWLoriT	pSARP
UWLS-R	GCAGACACCAGGTCGATCCTACCAACCGGCACGATTGTCCA	pUWLoriT	pSARP
SARPg-F	CCGGTTGGTAGGATCGACCTGGTGTCTGCTGTCGGCCG	*A. fjordicus* ADI127-17 gDNA	pSARP
SARPg-R	GAGTGCACCATACGCATTCGGCTGCACCACGTCGC	*A. fjordicus* ADI127-17 gDNA	pSARP
809-127-F	GCATCGAGCGGTCCTGCAGGTCGACTCTAGAGGATCCGCG	pSOK809	pA127-LP
809-127-R	GCAGACACCAGGTCGGTGAACTCTCCTTCGATCGATGGACAGC	pSOK809	pA127-LP
A127-LP-F	TCGAAGGAGAGTTCACCGACCTGGTGTCTGCTGTCGGCCG	*A. fjordicus* ADI127-17 gDNA	pA127-LP
A127-LP-R	AGTCGACCTGCAGGACCGCTCGATGCAGCGAAGGC	*A. fjordicus* ADI127-17 gDNA	pA127-LP
809-94011-F	CCAGGCGGGACGTGCAGGTCGACTCTAGAGGATCCG	pSOK809	p9401-LP1
809-94011-R	GTCTCACCTGCTCGTGAACTCTCCTTCGATCGATGGACAG	pSOK809	p9401-LP1
94011-LP-F	TCGATCGAAGGAGAGTTCACGAGCAGGTGAGACGAACATGGAGACGAACG	*Streptomyces* sp. MP94-01 gDNA	p9401-LP1
94011-LP-R	CCTCTAGAGTCGACCTGCACGTCCCGCCTGGCCGGGTCCG	*Streptomyces* sp. MP94-01 gDNA	p9401-LP1

809-94012-F	TCGATCGAAGGAGAGTTCACCAGCTGA TGCCCCTGACCCGAG	pSOK809	p9401-LP2
809-94012-R	CCTCTAGAGTCGACCTGCAAGAGGCTGT GGGGGTCCC	pSOK809	p9401-LP2
94012-LP-F	TGCAGGTCGACTCTAGAGGAT	*Streptomyces* sp. MP94-01 gDNA	p9401-LP2
94012-LP-R	GTGAACTCTCCTTCGATCGATG	*Streptomyces* sp. MP94-01 gDNA	p9401-LP2
809-9810-F	GGCGGCATGAGTGTGCAGGTCGACTCT AGAGGATCCGC	pSOK809	p9819-LP
809-9810-R	CGGTTGACGACCGTGAACTCTCCTTCGA TCGATGGACAGC	pSOK809	p9819-LP
9810-LP-F	CGAAGGAGAGTTCACGGTCGTCAACCG TACAGAAGGGA	*Streptomyces* sp. MP98-10 gDNA	p9819-LP
9810-LP-R	GAGTCGACCTGCACACTCATGCCGCCCC GCA	*Streptomyces* sp. MP98-10 gDNA	p9819-LP
809Sven-F	CCTTGGCCACGACCTGCAGGTCGACTCT AGAGGATCCG	pSOK809	pSVEN-LP
809Sven-R	GGTCGGTACGGCAACGGTGAACTCTCCT TCGATCGATGGACAGC	pSOK809	pSVEN-LP
Sven-LP-F	AGGAGAGTTCACCGTTGCCGTACCGAC CACAGAGAGG	*S. venezuelae* ATCC 10712 gDNA	pSVEN-LP
Sven-LP-R	AGTCGACCTGCAGGTCGTGGCCAAGGT CGACCGC	*S. venezuelae* ATCC 10712 gDNA	pSVEN-LP
809-Snou-F	CCATCGACCTGTTGCTGCAGGTCGACTC TAGAGGATCCGCG	pSOK809	pSNOU-LP
809-Snou-R	CCGGTACGGAAGGTTCGGTGAACTCTCC TTCGATCGATGGACAGC	pSOK809	pSNOU-LP
Snou-LP-F	AGGAGAGTTCACCGAACCTTCCGTACC GGAACGCGT	*S. noursei* ATCC 11455 gDNA	pSNOU-LP
Snou-LP-R	AGTCGACCTGCAGCAACAGGTCGATGG CTTCTTGCCTGGT	*S. noursei* ATCC 11455 gDNA	pSNOU-LP
p9401_1_dA-C-HindIII-P1[#]	GACTGGTTTGCGGGAACCGCCTGGATCT GCTGATCCGACAAGCTTGTGTAGGCTGG AGCTGCTTC	pTOPO_Kan_ermEp	P9401-LP1-ermEp*
p9401_1_dA-C-NdeI-R[#]	CGGCGCGGCCGGGCTCTCGGGCAACAC GACGAAATCCATATGCTCGAGGGGGGG CCCGATCCTACC	pTOPO_Kan_ermEp	P9401-LP1-ermEp*
hrdB-F	CCAAGGGCTACAAGTTCTCC	cDNA	
hrdB-R	CGCGAGCTTGTTGATGACC	cDNA	
A127-LP-A-F	GTGACGGAGTCGCTCGGATCG	cDNA	
A127-LP-A-R	ACCAGTCGGCCTCGAATCCCC	cDNA	
A127-LP-C-F	CGGACGATGAGGTCTGCTGGG	cDNA	
A127-LP-C-R	GGACAGTGTGCCCTGTGCCTG	cDNA	
9401-LP2-A-F	CGTACCGTCCCTCGAGGAACC	cDNA	
9401-LP2-A-R	GTACTGGGCATTGGCGTCGG	cDNA	
9401-LP2-C-F	ACCTGCGCTGCCCGTCGCCTG	cDNA	
9401-LP2-C-R	CCAGAGCAGGGGTTCGCCTG	cDNA	

[#] primers for modification of p9401-LP1 by PCR targeting

Table S3. Size and product accession number of core biosynthetic genes. bp: base-pair.

Name	A (precursor)	C (macrolactam synthetase)	E (RiPP recognition element)	B (cysteine protease)
A127-LP	144 bp (ARA91552.1)	1827 bp (ARA91553.1)	258 bp (ARA91554.1)	414 bp (ARA91555.1)
9401-LP1	126 bp (ARA91539.1)	1842 bp (ARA91540.1)	261 bp (ARA91541.1)	429 bp (ARA91542.1)
9401-LP2	144 bp (ARA91543.1)	1902 bp (ARA91544.1)	894 bp (ARA91545.1)	
9810-LP	123 bp (ARA91546.1)	1920 bp (ARA91547.1)	252 bp (ARA91548.1)	456 bp (ARA91549.1)
Snou-LP	150 bp (ARA91559.1)	1821 bp (ARA91560.1)	261 bp (ARA91561.1)	426 bp (ARA91562.1)
Sven-LP	132 bp (ARA91563.1)	1806 bp (ARA91564.1)	555 bp (ARA91565.1)	

Table S4. Calculated and measured *m/z* of the new lasso peptides.

Lasso peptide	Ions detected	Calculated monoisotopic *m/z*	Experimental monoisotopic *m/z*	Error (ppm)
9401-LP1	$[M+2H]^{2+}$	983.9162	983.9165	-0.30
9810-LP	$[M+3H]^{3+}$	669.3476	669.3479	-0.45
Snou-LP	$[M+2H]^{2+}$<br>$[M+3H]^{3+}$	1123.5389<br>749.3617	1123.5374<br>749.3617	1.34<br>0.00

Table S5. IM-MS data generated in this study.

	**$[M+2H]^{2+}$**		**$[M+3H]^{3+}$**		**$[M+4H]^{4+}$**		Monoisotopic molecular mass	ζ (e.kDa^{-1})	$\Delta\Omega$ / Ω (%)
Peptide	Drift Time (ms)	Rel. Int. (%)	Drift Time (ms)	Rel. Int. (%)	Drift Time (ms)	Rel. Int. (%)			
MccJ25	8.68	0.03	4.77	100.00	3.42/3.96	22.84	2106.02	1.51	7.68
MccJ25-lcm	9.30	0.55	5.80/5.15	23.12	4.88	100.00	2106.02	1.81	23.55
9401-LP1	8.68/8.19	22.00	4.45	100.00	-	-	1965.82	1.67	5.58
Snou-LP	-	-	5.15	34.01	3.63	100.00	2245.06	1.43	3.38

Figure S1. LC-MS analysis of the native (A) and reduced 9401-LP1 (B). Extracted ion chromatograms and mass spectra are shown.

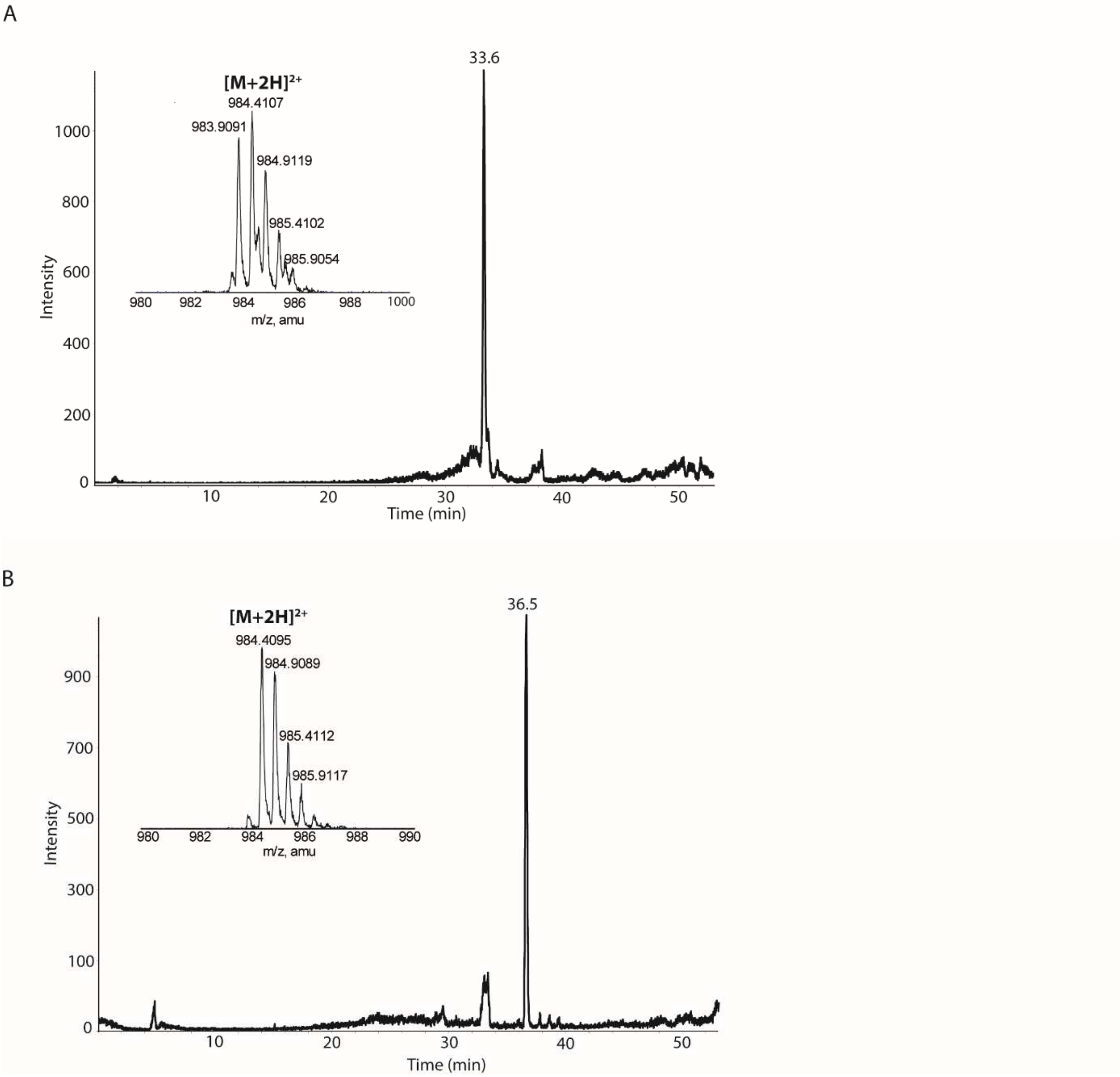

Figure S2. Hydrolysis of a control non-lasso peptide by carboxypeptidase Y treatment. The peptide used is the topoisomer of microcin J25, MccJ25-lcm (sequence GGAGHVPEYFVGIGTPISFYG, macrolactam ring involving an isopeptidic bond between G1 and E8) (Ducasse et al. 2012). **(A)** Extracted ion chromatograms obtained after incubation of MccJ25-lcm in the absence (blue) or in the presence (red) of carboxypeptidase Y for the intact peptide ($[M+2H]^{2+}$ species at *m/z* 1054.0) and for the hydrolysis product [G1-Y9] ($[M+2H]^{2+}$ at *m/z* 434.7). **(B)** MS spectrum of [G1-Y9] **(C)** MS/MS spectrum of [G1-Y9] ($[M+2H]^{2+}$ at *m/z* 434.7, collision energy 44 eV).

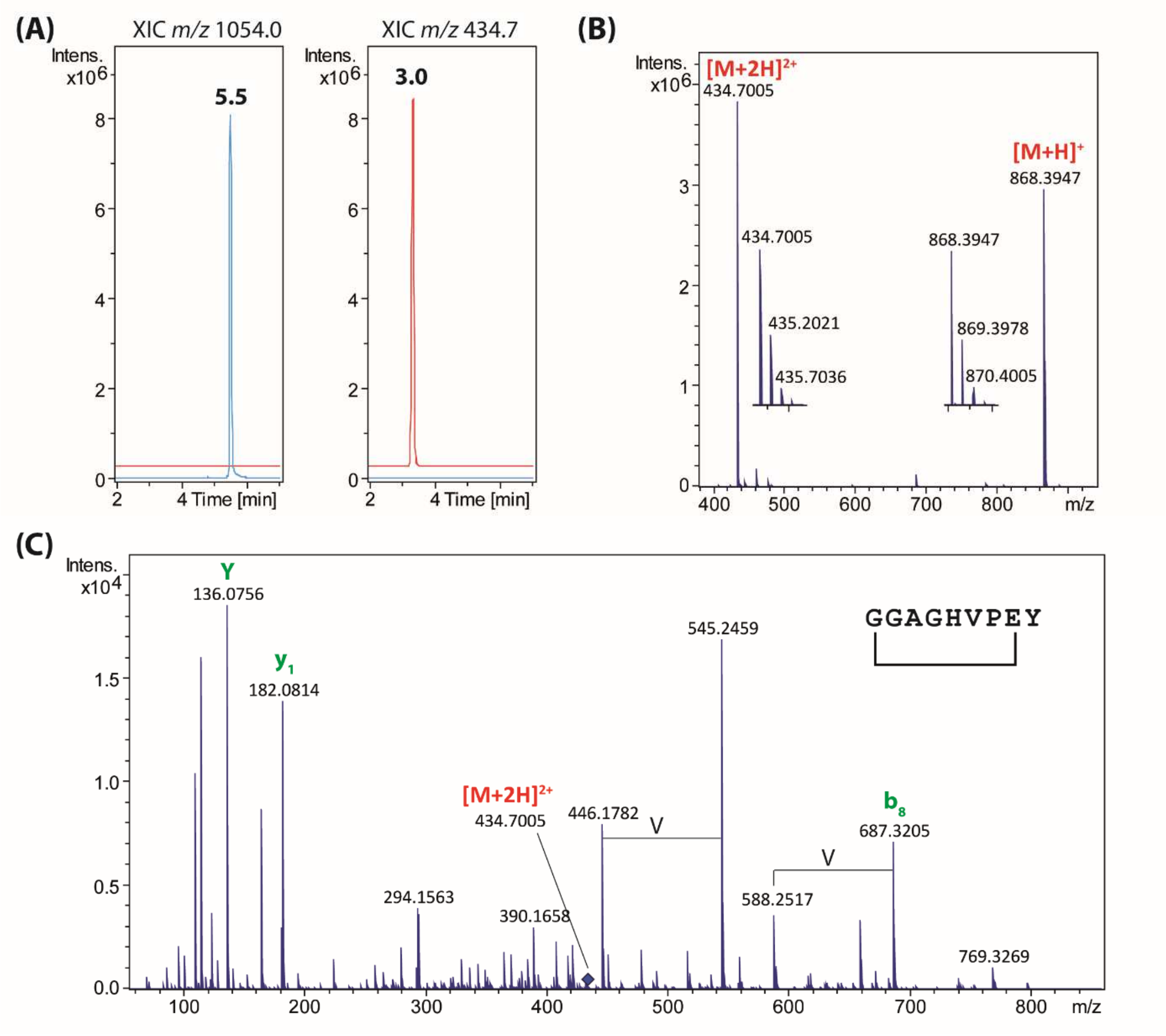

Figure S3. Partial resistance of 9401-LP1 to carboxypeptidase Y digestion. Hydrolysis was achieved in under reducing and non-reducing conditions. Two hydrolysis products were obtained in reducing conditions: (A) [A1-I17] and (B) [A1-W10]. From left to right are shown the extracted ions chromatograms [incubation of 9401-LP1 alone (blue), in the presence of carboxypeptidase Y (green), in the presence of carboxypeptidase Y and DTT (red)], and the MS and MS/MS spectra of the hydrolysis products. Note that due to co-elution with an isobaric contaminant, the MS/MS spectra of [A1-I17] could not be obtained with satisfying quality. The MS/MS spectra of [A1-W10] is shown for the $[M+H]^+$ species (*m/z* 1119.5, collision energy 55 V).

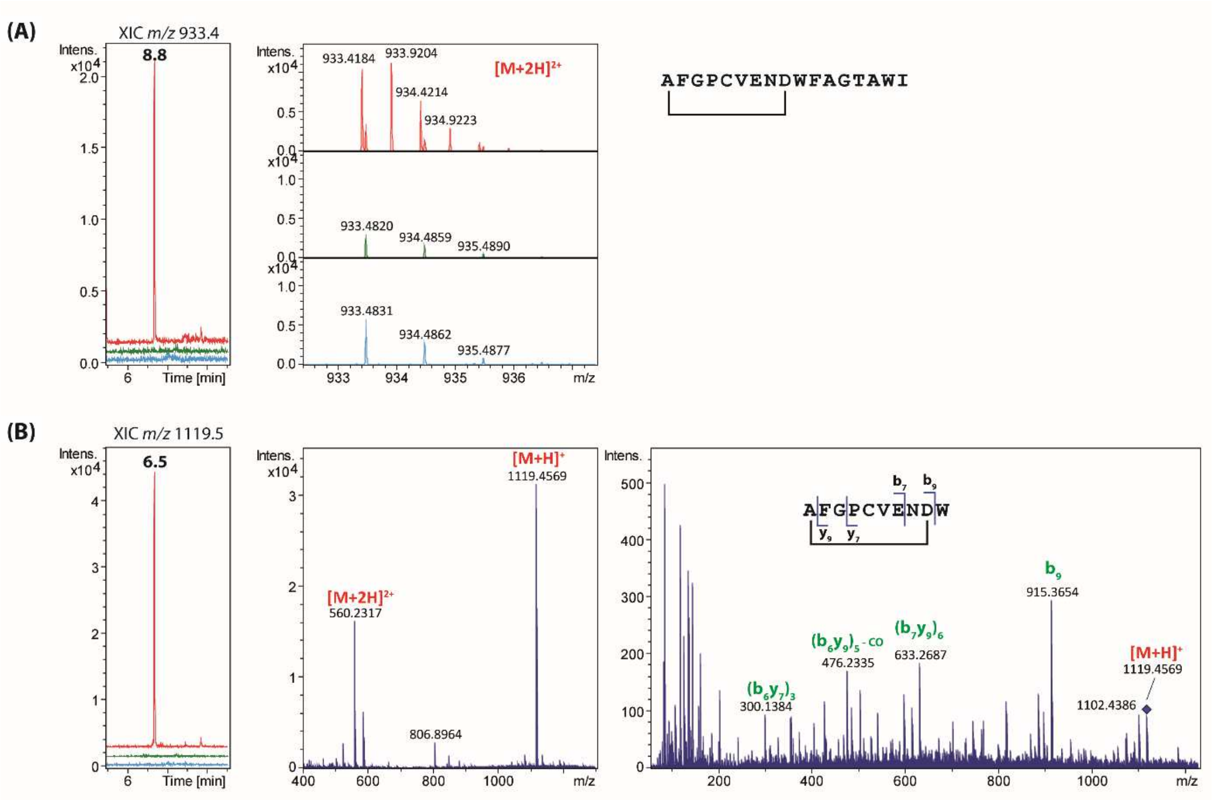

Figure S4. Partial resistance of 9810-LP to carboxypeptidase Y digestion. (A) Extracted ion chromatograms obtained after incubation of 9810-LP in the absence (blue) or in the presence (red) of carboxypeptidase Y for the intact peptide ($[M+3H]^{3+}$ species at *m/z* 669.3) and for the hydrolysis products [G1-I15] ($[M+3H]^{3+}$ at *m/z* 636.3), [G1-R14] ($[M+3H]^{3+}$ at *m/z* 598.6) and [G1-Y10] ($[M+2H]^{2+}$ at *m/z* 597.8). (B-D) MS and MS/MS spectra of the hydrolysis products.

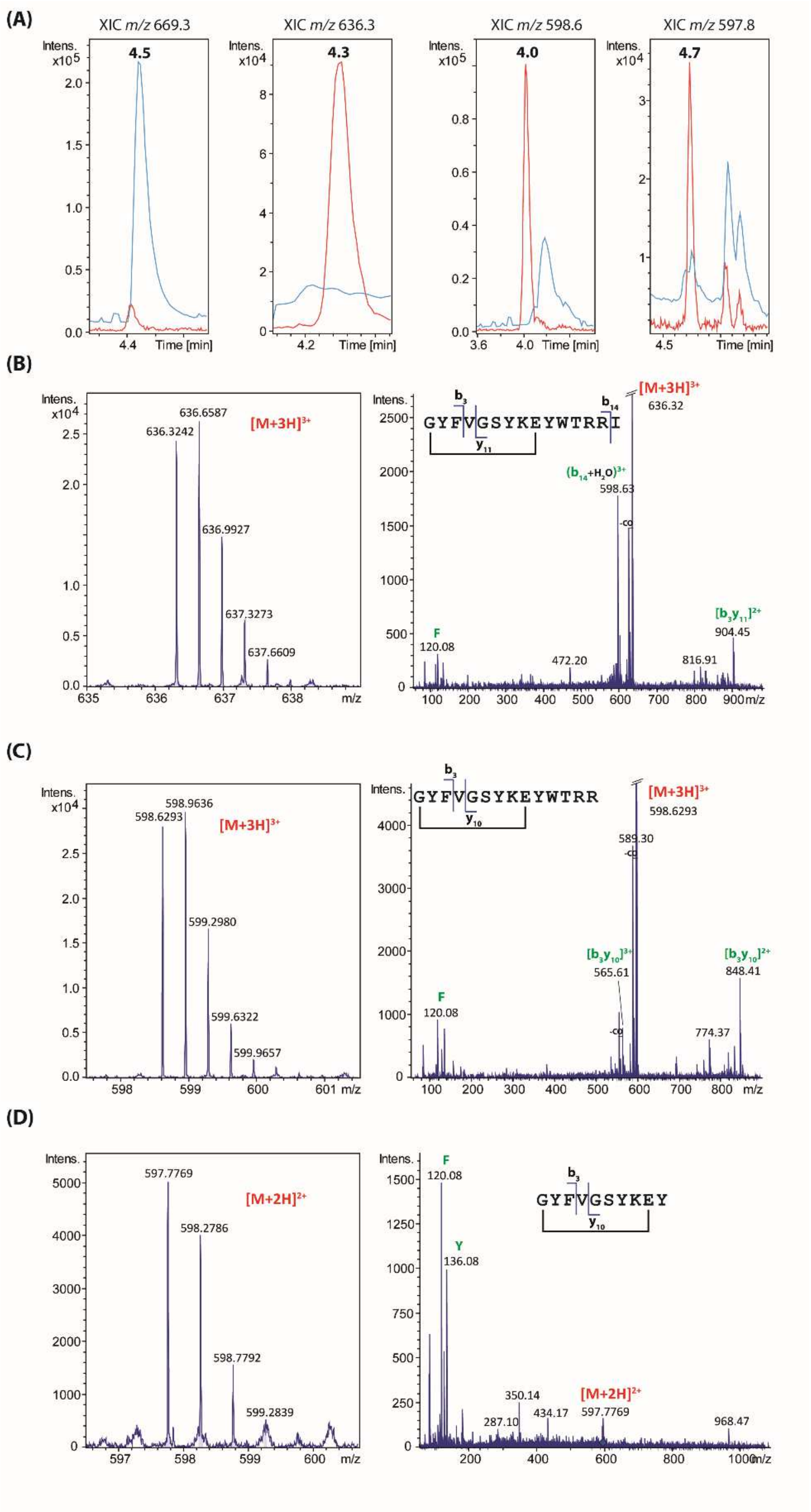

Figure S5. Partial resistance of Snou-LP to carboxypeptidase Y digestion. Extracted ion chromatograms obtained after incubation of Snou-LP in the absence (blue) or in the presence (red) of carboxypeptidase Y (A) for the intact peptide ($[M+3H]^{3+}$ species at *m/z* 749.4) and (B) for the hydrolysis product [Y1-N9] ($[M+H]^{+}$ at *m/z* 1045.5). (C) MS spectrum of [Y1-N9].

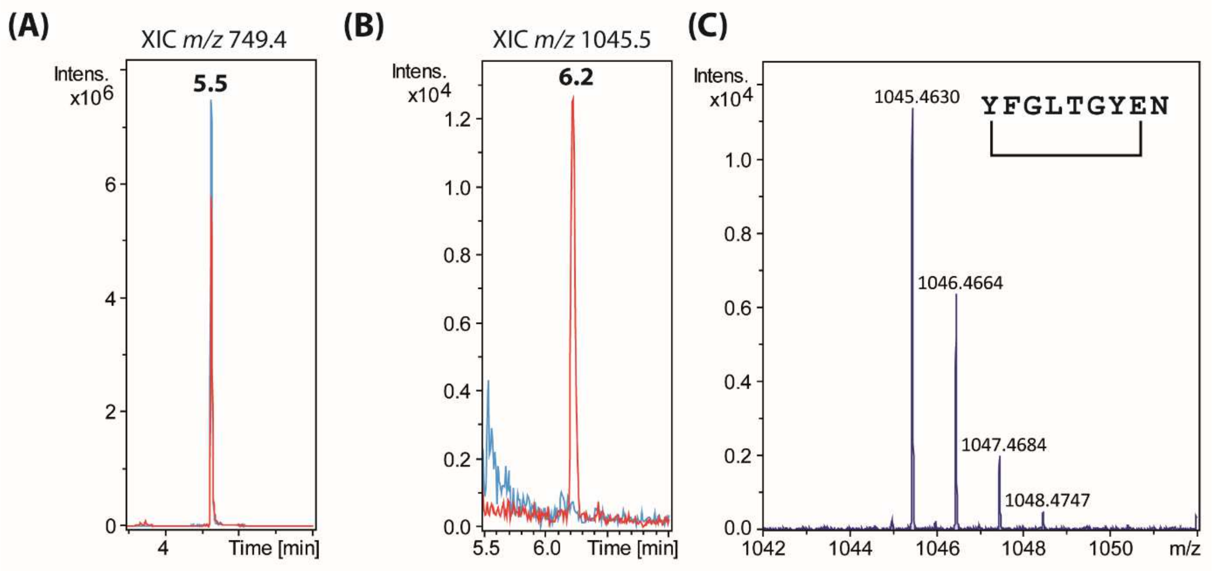

Figure S6. Drift time profiles of peptides 9401-LP1 and Snou-LP obtained by IM-MS. Extracted ion mobility profiles and mass spectra of the $[M+3H]^{3+}$ (*m/z* 656.3) and $[M+2H]^{2+}$ (*m/z* 983.9) species of 9401-LP1 (A,B) and of the $[M+4H]^{4+}$ (*m/z* 562.3) and $[M+3H]^{3+}$ (*m/z* 749.4) species of Snou-LP (C,D).

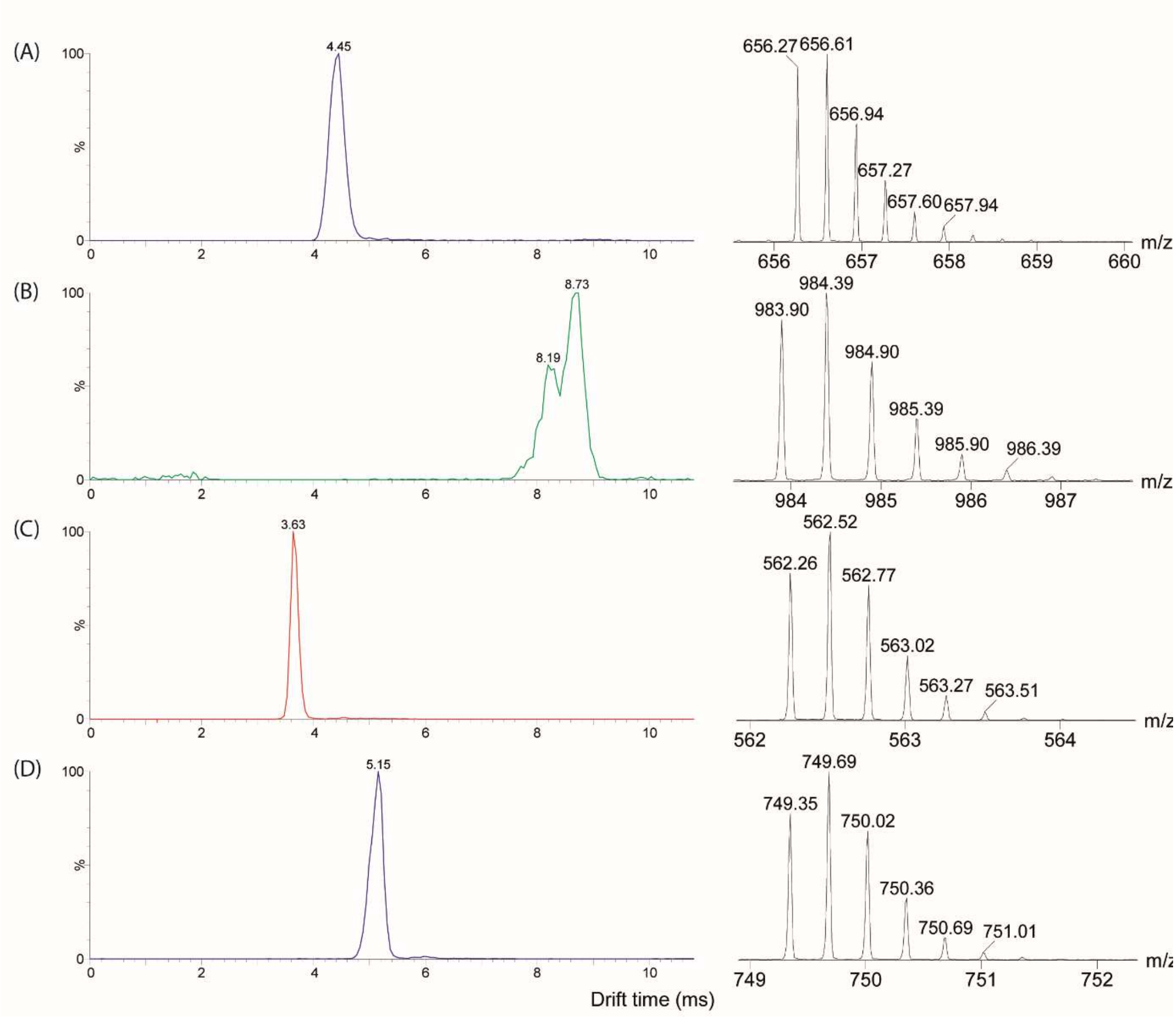

Figure S7. Plot representing two IM-MS indicators of the lasso topology for peptides 9401-LP1 and Snou-LP, in comparison with a collection of lasso and non-lasso peptides. The ratio ΔΩ/Ω (%) measures the relative range of CCS covered by all charge states while ζ is the mean charge divided by mass (Jeanne Dit Fouque K et al. 2017) The peptides analyzed in this study are circled. They consist of the lasso peptide MccJ25 together with its non-lasso topoisomer MccJ25-lcm, 9401-LP1 and Snou-LP. The other points were generated in a previous study (Jeanne Dit Fouque K et al. 2017). Note that the control peptides MccJ25 and MccJ25-lcm repeated in the two independent studies showed the same tendencies. The peptides are colored according to their type: non-lasso peptides in red, type II lasso peptide in dark blue, type I lasso peptides in light blue and type III lasso peptides in orange.

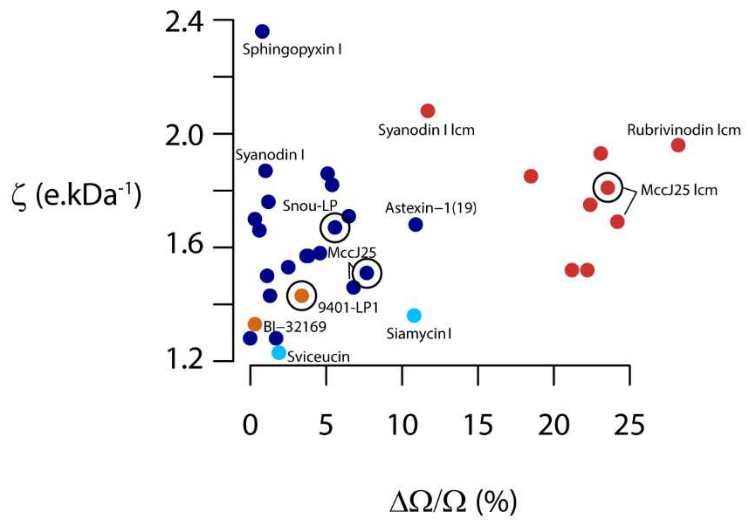

Figure S8. Modification of the 9401-LP1 cluster. Color code for genes: red (A encoding the precursor), blue (C encoding the macrolactam synthetase), orange (E encoding the RiPP recognition element), yellow (B encoding the protease) and green (D encoding the ABC transporter).

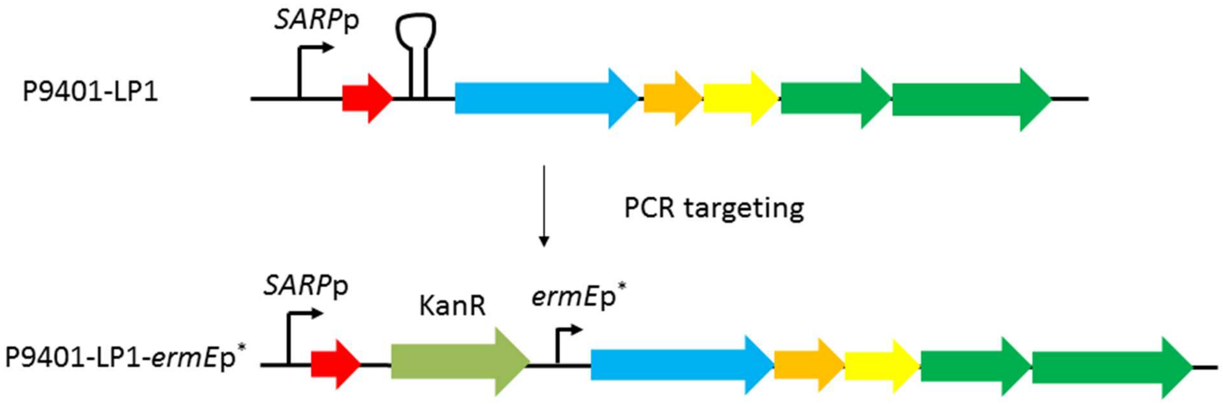

Figure S9. Production level of modified 9401-LP1 construct in *S. albus*. EIC: extracted ion chromatograms. Blue/red/orange lines: three clones with p9401-LP1-ermEp*; green/purple lines: two clones with p9401-LP1 native cluster.

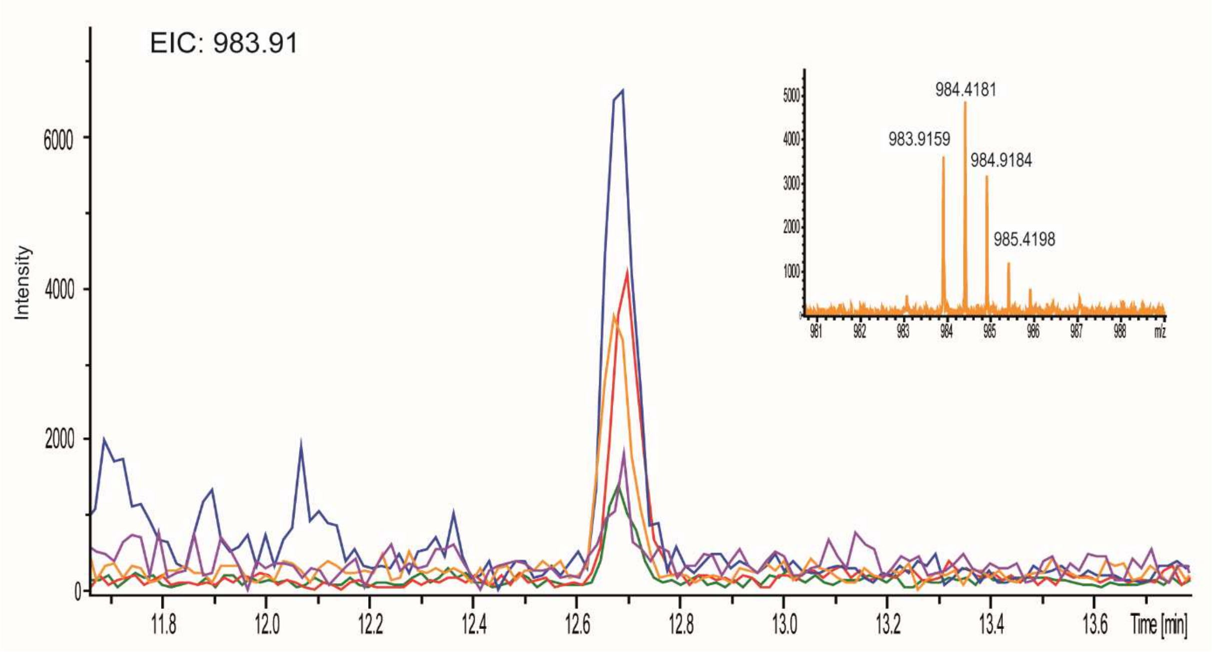

Figure S10. Confirmation of gene expression of A127-LP (A) and 9401-LP2 (B) clusters by RT-PCR. RT0: no RNA template control; RT: cDNA; RT-: no reverse transcriptase control. Expected fragment size: *hsrB* (120 bp), A127-LP-A (106 bp), A127-LP-C (203 bp), 9401-LP2-A (121 bp), 9401-LP2-C (128 bp). ***** denotes the correct band corresponding to the 9401-LP2-C amplicon.

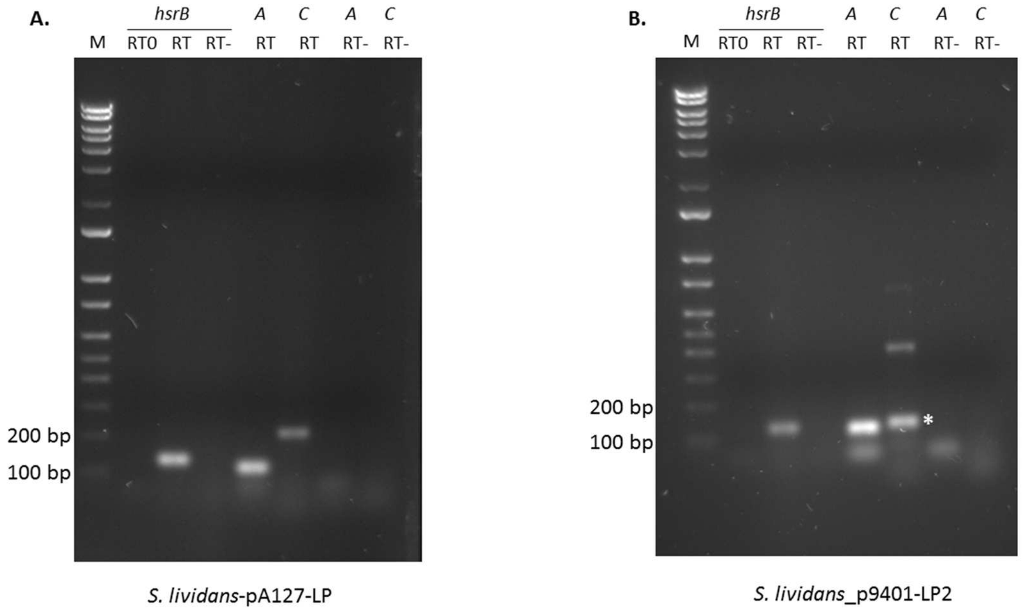

References

1. Chater, K. F., and Wilde, L. C. (1976) Restriction of a bacteriophage of Streptomyces albus G involving endonuclease SalI. J Bacteriol 128, 644-650.
2. Ducasse, R., Yan, K. P., Goulard, C., Blond, A., Li, Y., Lescop, E., Guittet, E., Rebuffat, S., Zirah, S. (2012) Sequence determinants governing the topology and biological activity of a lasso peptide, microcin J25. Chembiochem. 13, 371-80.
3. Gust, B., Challis, G. L., Fowler, K., Kieser, T., and Chater, K. F. (2003) PCR-targeted Streptomyces gene replacement identifies a protein domain needed for biosynthesis of the sesquiterpene soil odor geosmin. *Proc Nat Acad Sci* 100, 1541-1546.
4. Gomez-Escribano, J. P., and Bibb, M. J. (2011) Engineering Streptomyces coelicolor for heterologous expression of secondary metabolite gene clusters. *Microb Biotechnol* 4, 207-215.
5. Jeanne Dit Fouque, K., Lavanant, H., Zirah, S., Hegemann, J. D., Zimmermann, M., Marahiel, M. A., Rebuffat, S. and Afonso, C. (2017) Signatures of Mechanically Interlocked Topology of Lasso Peptides by Ion Mobility–Mass Spectrometry: Lessons from a Collection of Representatives. J Am Soc Mass Spectrom. 28, 315-322.
6. Rückert, C., Albersmeier, A., Busche, T., Jaenicke, S., Winkler, A., Friðjónsson, Ó. H., Hreggviðsson, G. Ó., Lambert, C., Badcock, D., Bernaerts, K., Anne, J., Economou, A., and Kalinowski, J. (2015) Complete genome sequence of Streptomyces lividans TK24. *J Biotech 199*, 21-22.
